# From spinodal decomposition to alternating layered structure within single crystals of biogenic magnesium calcite

**DOI:** 10.1038/s41467-019-12168-8

**Published:** 2019-10-08

**Authors:** Eva Seknazi, Stas Kozachkevich, Iryna Polishchuk, Nuphar Bianco Stein, Julie Villanova, Jussi-Petteri Suuronen, Catherine Dejoie, Paul Zaslansky, Alex Katsman, Boaz Pokroy

**Affiliations:** 1 Department of Materials Science and Engineering and the Russel Berrie Nanotechnology Institute, Technion-Israel Institute of Technology, 32000 Haifa, Israel; 2 ESRF-The European Synchrotron Radiation Facility, CS 40220, 38043 Grenoble Cedex 9, France; 3 Department for Restorative and Preventive Dentistry, Centrum für Zahn-, Mund- und Kieferheilkunde, Charité–Universitätsmedizin Berlin, 14197 Berlin, Germany

**Keywords:** Bioinspired materials, Biomineralization, Materials science

## Abstract

As organisms can form crystals only under ambient conditions, they demonstrate fascinating strategies to overcome this limitation. Recently, we reported a previously unknown biostrategy for toughening brittle calcite crystals, using coherently incorporated Mg-rich nanoprecipitates arranged in a layered manner in the lenses of a brittle star, *Ophiocoma wendtii*. Here we propose the mechanisms of formation of this functional hierarchical structure under conditions of ambient temperature and limited solid diffusion. We propose that formation proceeds via a spinodal decomposition of a liquid or gel-like magnesium amorphous calcium carbonate (Mg-ACC) precursor into Mg-rich nanoparticles and a Mg-depleted amorphous matrix. In a second step, crystallization of the decomposed amorphous precursor leads to the formation of high-Mg particle-rich layers. The model is supported by our experimental results in synthetic systems. These insights have significant implications for fundamental understanding of the role of Mg-ACC material transformation during crystallization and its subsequent stability.

## Abstract (web summary)

Several fields are interested in the mechanism by which organisms control crystallisation at ambient temperatures. Here, the authors evaluate the mechanism of magnesium rich nanoprecipitate layer formation in the calcite crystals of the brittle star, via the decomposition of the amorphous precursor.

## Introduction

Biominerals are minerals that originate from biological processes. Although formed under ambient conditions and from only a limited variety of elements, biominerals can exhibit outstanding properties related to their specific functions^1–6^. For example, the calcitic teeth of the sea urchin are composed of brittle calcite, yet they exhibit mechanical properties optimized to serve their grinding function^3,4^. Likewise, the lenses of the brittle star possess optimized optical properties despite being composed of calcite, a birefringent material^5^. Considerable research has focused on these biominerals from the perspective of materials science, in an attempt to understand the specific compositions and structures responsible for their superior properties^7–9^, and to learn strategies that can be applied in the design of novel synthetic materials^10–15^.

Research on marine biominerals has highlighted recurrent strategies for enhancement of mechanical properties. Common strategies include the use of hierarchical structures^8,10^, exploiting the presence of intra- and inter-crystalline organic molecules^1,2,16^, and substitution of Mg for Ca in calcitic biominerals^3,4,6,9^. Recently, Polishchuk et al. uncovered a strategy that involves a complex distribution of Mg in calcite, and which acts as a toughening mechanism in the lenses of the brittle star *Ophiocoma wendtii*^6^.

The brittle star is an echinoderm that possesses several skeletal parts^17^, including lenses, spicules, teeth, and arm vertebrae, which, respectively, serve its visual system^5^, locomotor system, oral system, and act as joints^18^. A study of the lens nanostructure showed that each lens comprises a single crystal composed of two coherent Mg-calcite phases: a phase comprising nanoparticles of high-Mg calcite (~5 nm diameter, ~40 mol% MgCO_3_), coherently incorporated in a phase consisting of a poorer Mg-calcite matrix (~13 mol% MgCO_3_)^6^. It was further shown that the nanoprecipitates are not dispersed homogeneously within the single-crystalline lenses, but are organized in layers, parallel to the surface, that are alternately richer and poorer in Mg-nanoinclusions^6^. These two nanostructural features—the existence of coherent Mg-rich nanoinclusions and their layered distribution—improve lens toughness (increasing it by three-fold compared to geological calcite)^6^. Together, the existing coherence and the lattice mismatch between the Mg-rich nanoinclusions and their surrounding matrix induce compressive stresses in the matrix, creating a prestressed material with consequently improved hardness and fracture toughness^6^. Moreover, the parallel arched layers within the lenses create a structure with alternating compressive stresses and elastic modulus, promoting crack deflection and thus further enhancing their toughness^6^. As pointed out by Duffy^19^, this discovered strategy may represent the solution to the dual need of optimizing the mechanical function of the lens without compromising its optical function.

In this work, utilizing synchrotron radiation and electron microscopy techniques, we characterized the layered structure of the brittle star lenses and spicules. We also addressed the remaining fundamental questions concerning the mechanisms of formation of this complex, highly functional nanostructured material under ambient conditions. We propose a formation scenario of the brittle star lens nanostructure, involving spinodal decomposition of the amorphous supersaturated precursor followed by crystallization from a single nucleation event. To support the proposed biological route of formation we present experimental results that indicate that in synthetic Mg amorphous calcium carbonate (Mg-ACC) a spinodal decomposition indeed precedes crystallization of higher and lower Mg-calcite phases. We also show that organics suppress the spinodal decomposition and that most probably the paucity of organics present in the brittle star’s mineralized tissue allows it to occur in this system. Unraveling the mechanism of formation of the brittle star’s calcite biomineral has important implications for understanding the role of the Mg-ACC material prior to and during crystallization and for the success of synthetically growing various single crystals at ambient temperatures, which is not possible as yet.

## Results

### The layered structure

**Fig. 1:**
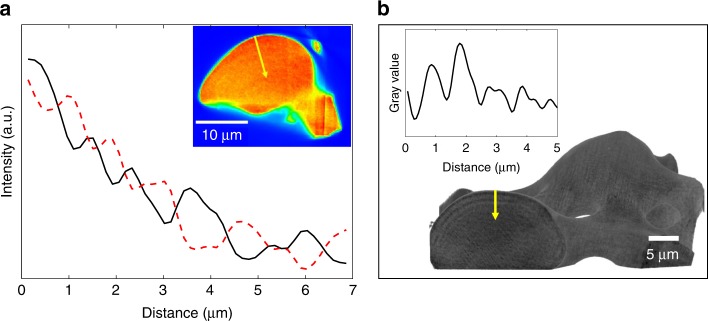
The layered structure revealed by tomography. **a** XRF tomography reconstruction slice across a lens (pixel size is 150 nm, red areas correspond to higher Ca content). Corresponding intensity line plot taken from the lens surface towards the center of the bulk (marked by a yellow arrow), shows the measured oscillations of the calcium signal (black) compared with theoretical schematic oscillations of the magnesium content (dashed red), perpendicular to the sample surface. **b** Typical nano-HT reconstructed dorsal arm plate (DAP) part, showing oscillations in mass-density (pixel size is 35 nm). Corresponding intensity line plot taken from the lens surface towards the center of the bulk (marked by a yellow arrow)

Our previous study of the nanostructure of brittle star lenses provided strong evidence for the presence of arched layers oriented parallel to the lens surface^6^. Among others, small-angle X-ray scattering (SAXS) measurements indicated a layered distribution of few nm-sized features (periodicity of ~2.5–16.5 nm). In quest to further understand what these layers are comprised of, we performed additional characterization utilizing several techniques. Firstly, we used synchrotron X-ray fluorescence (XRF) tomography obtained by a nanobeam in the synchrotron to map the elemental distribution of the lenses. A typical reconstruction map of Ca distribution (Fig. 1a, inset) and the corresponding intensity line plots (Fig. 1a) reveal intensity oscillations of this specific element.

Oscillation of the Ca signal correlates well with the existence of alternating Ca-depleted and Ca-rich layers beneath the lens surface. We could not obtain a signal for Mg due to energy detection restriction (the energy of the K-alpha radiation is too low and absorbed by air surrounding the sample during measurement). Nevertheless, since Mg substitutes for Ca in a calcite lattice and is by far the most abundant impurity detected within the lenses (as determined from ICP-OES by Polishchuk et al.^6^), it is reasonable to predict that the distribution of Mg is complementary to that of Ca, as represented by the dashed red line in Fig. 1a. Besides the oscillations, the signal undergoes a decrease from the sample’s surface toward the bulk, which can be attributed to sample self-absorption. Moreover, the oscillations, although irregular, demonstrate a periodicity of 0.7−1.5 µm. These periodicity values coincide with the values determined from the nano-holotomographic (nano-HT) reconstruction of a slice from another lens sample (Fig. 1b). In a previous study by Dai et al.^20^, where Mg-calcite was crystalized from polymer-induced liquid precursor (PILP), transition bars were observed and shown to be rich in the biopolymer that stabilizes the PILP. In light of this, we considered whether our layers are comprised of organics as well. The first experiment we performed to answer this question was recharacterization of the lenses by nano-HT after heat treatment for 70 min at 300 °C. Interestingly, same oscillations were observed in the nano-HT reconstruction of a lens as compared to those without annealing (Supplementary Fig. 1). We want to stress that the annealing temperature is sufficiently high for occluded proteins to decompose, as we have shown extensively in the past^2,21,22^, confirming that organic matter could not be solely responsible for the observed layered structure. Another strong indication that eliminates the possibility that the observed layers are comprised of higher organic concentration is the fact that these layers demonstrate smaller d-spacings than the matrix, as we have previously shown^6^. This therefore contradicts the possibility of higher concentration of intracrystalline organics within the layers, which have been shown to induce expansion of the d-spacings rather than contraction^2,21,22^. In this context, we measured the amount of occluded proteins in the brittle star lenses by means of amino acids analysis (AAA). We found that the brittle star lenses contain as little as 0.001 wt% of occluded proteins, as determined by AAA (Supplementary Table 1) (for comparison, the mature sea urchin spines contain 0.02 wt% occluded proteins^23^). This extremely low amount, together with the other above characterization results, clearly imply that the distribution of Mg-rich nanoprecipitates (rather than the presence of proteins) is the most probable cause for the observed layered structure, although we do not rule out some possible synergistic effect involving organic matter in the formation of the layers.

**Fig. 2:**
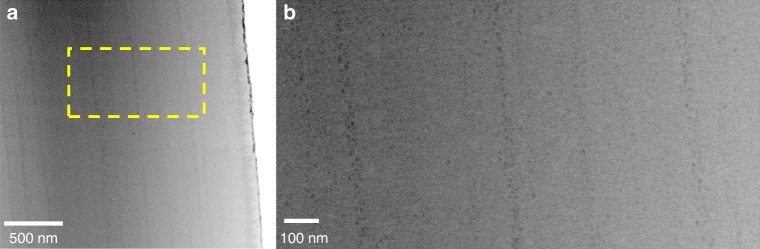
The layered structure is caused by a layered distribution of high-Mg nanoparticles. **a** HRTEM (HAADF-STEM mode) of a FIB-cut lens lamella (cut perpendicularly to a lens surface–the lens surface can be seen at the right edge of the image) showing the existence of light (dark) nanoparticles distributed in layers parallel to the surface. **b** Zoom-in corresponding to the yellow square in (**a**)

We could confirm that the observed layered structure corresponds to a layered distribution of high Mg-particles using high-resolution transmission electron microscopy (HRTEM). Indeed, imaging of a lens’ cross-section clearly shows a layered distribution of nanoparticles (Fig. 2). The image of Fig. 2 was acquired in high-angle annular dark-field scanning transmission electron microscopy mode (HAADF-STEM), which is sensitive to elemental composition (Z-contrast). The fact that the nanoparticles are darker contrast than the matrix implies that they are lighter, corresponding to the fact that they are richer in Mg. Therefore, the particles seen in Fig. 2 correspond to the previously established coherent high Mg-nanoparticles, and they are organized in layers parallel to the surface. The particle-rich layers are irregularly spaced and irregularly packed with particles, explaining the irregular line profiles obtained with the different characterization techniques (Fig. 1, Supplementary Figs. 1, 2 and 4).

We would like to note that the difference in appearance of the layered structure between the different characterization techniques is due to the difference in length scales that they characterize. Indeed, the small thickness of the particle-rich layers (few tens of nm) as seen in HRTEM cannot be imaged and detected in lower resolution techniques, and the micron-scale oscillations as seen in tomography are integrations of several thin layers, and cannot be detected at the high magnification used in HRTEM.

**Fig. 3:**
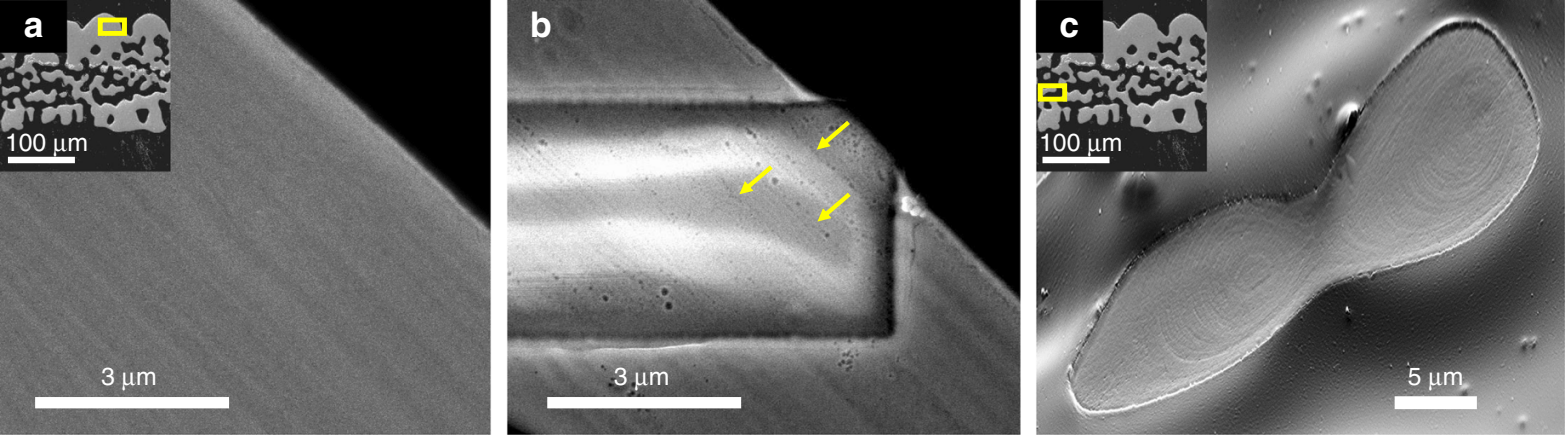
The layered structure revealed by electron microscopy **a** HRSEM (BSE detector) image of a polished lens section. **b** BSE image of the same specimen after ion-milling in the FIB. Yellow arrows point at visible layers in the milled box. **c** HRSEM (secondary electron (SE) detector) images from polished DAP. The insets in **a** and **c** are a low magnification image of the polished DAP, the yellow rectangles represent the corresponding areas of interest

Additional evidence for the layered structure emerges when employing electron microscopy using a Z-sensitive backscattered electron (BSE) detector. For this examination, we embedded DAPs into epoxy resin and polished them to expose a section perpendicular to the lenses’ surface. The acquired images indeed displayed oscillations in contrast, confirming the layered structure in the lenses (Fig. 3a). The stripes exhibiting darker contrast in Fig. 3 correspond to layers that comprise relatively higher concentrations of Mg-rich nanoparticles (therefore, less dense, being richer in Mg that is a lighter element as compared to Ca). On the other hand, the stripes with brighter contrast correspond to layers that comprise relatively lower concentrations of Mg-rich nanoparticles (denser, being less rich in Mg). To ensure that oscillations are present not only at the imaged surface but also in the bulk, we ion-milled an area of the sample using a focused ion beam (FIB). Continuing oscillations in density can be observed in the ion-milled portion (Fig. 3b). Since milling exposes a previously hidden surface, the observed oscillations are clear indications of the layered structure of the lenses, rather than the topography or the after-effects of sample preparation (polishing), a common concern in routine SEM preparations. The oscillations observed here are rather irregular (Fig. 3a, b) but range from 0.7 to 1.0 µm (Supplementary Fig. 2), confirming the data on periodicity obtained from the nano-HT experiment (Fig. 1).

High-resolution scanning electron microscope (HRSEM) images of all polished parts of the brittle star arm plates all show oscillations parallel to the surface (Fig. 3c), confirming the layered manner of its single-crystalline structure. In addition, the layered structure can be seen to appear as a series of periodic rings, probably resulting from the mechanism of its formation. The image in Fig. 3c was acquired using a secondary electron (SE) detector so the apparent contrast can originate from both topography and Z-contrast. We note that intensity oscillations originating from Z-contrast would attest for layers with different Mg-content, and oscillations originating from topography contrast in the polished sample would attest for layers with different polishing rate, which should be the case of layers with different content of Mg-rich particles. HRSEM image of a broken lens reveals similarly contrasting layers (Supplementary Fig. 3).

**Fig. 4:**
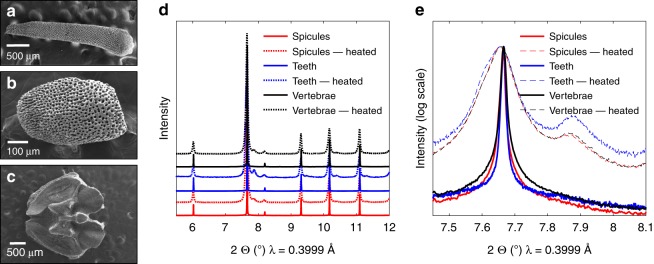
Other calcitic parts’ morphology and structure. **a**–**c** HRSEM images of the *O. wendtii* brittle star, showing **a** arm vertebrae, **b** spicules, and **c** teeth of the organism. **d** Diffractogram of these calcitic parts at room temperature and after heating treatment at 400 °C. **e** Zoom-in on the (104) diffraction peaks plotted on logarithmic scale

We found similar observations in other *O. wendtii* skeletal parts: spicules, arm vertebrae (both situated along the arms of the brittle star), and teeth (from the central ventral structure) (Fig. 4a–c). Synchrotron high-resolution powder X-ray diffraction (HRPXRD) patterns of the different crushed skeletal parts were identical and corresponded to single-phase Mg-calcite (Fig. 4d). In all elements, we observe the presence of the coherently aligned nanosized Mg-rich phase by demonstrating a broad hump at the base of the diffraction peaks plotted on the logarithmic scale and the emergence of a separate high-Mg calcite nanophase after the skeletal parts were heated at 400 °C (Fig. 4e). The lattice parameters of the different phases can be obtained from the diffractogram by means of Rietveld refinements, and the amount of Mg present in the calcite can then be quantified from the lattice parameters using previously established equations^24^ (Supplementary Table 2).

**Fig. 5:**
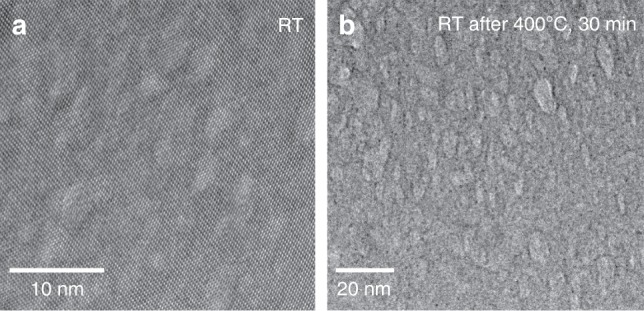
Coherent nanoparticles in the brittle star spicules. **a** HRTEM image of a spicule, revealing brighter nanodomains, which are coherent with the matrix. **b** HRTEM image after in-situ heating at 400 °C, demonstrating growth of the bright nanodomains and loss of coherency

Moreover, HRTEM imaging of an untreated section of a spicule shows a dark matrix with coherently incorporated bright nanodomains (Fig. 5a). These bright nanodomains grow and lose coherence after heat treatment at 400 °C (Fig. 5b), akin to the behavior demonstrated by Polishchuk et al. in the case of the lenses^6^. Based on these findings, we conclude that the different calcitic skeletal parts of the brittle star (lenses, spicules, arm vertebrae and teeth) all have the same nanostructure, consisting of Mg-rich nanoparticles (~40 mol% MgCO_3_) coherently embedded in a relatively lower Mg-calcite matrix (~13 mol% MgCO_3_), with resulting compressed matrix and overall improved toughness.

**Fig. 6:**
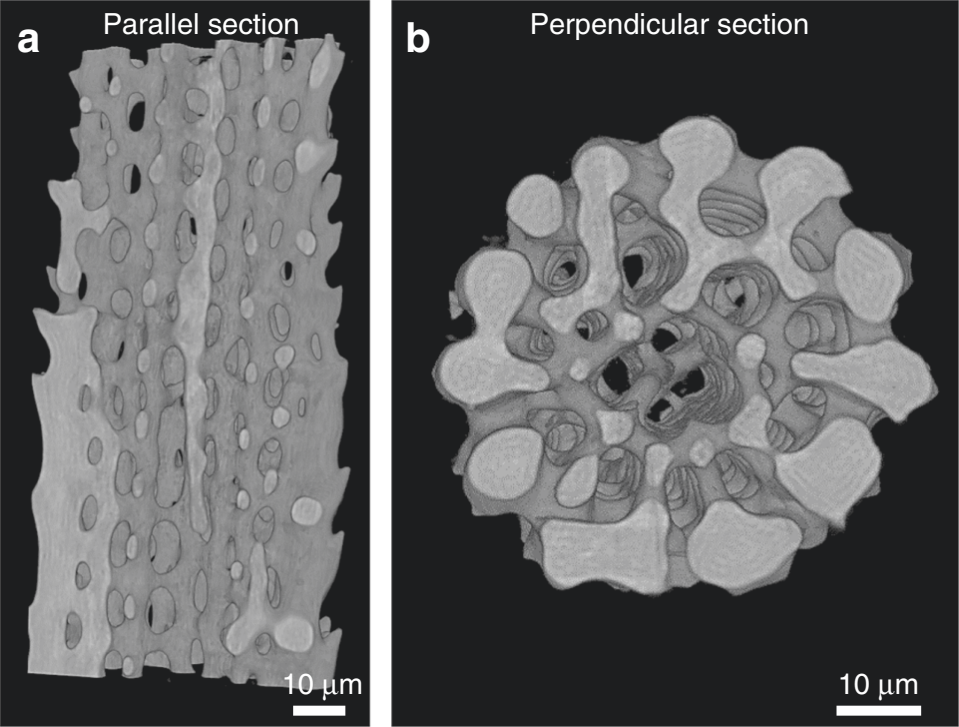
3D morphology and layered structure of spicules. Nano-HT reconstructed parallel section (**a**) and horizontal section (**b**) of the brittle star spicule (pixel size is 120 nm)

Nano-HT reconstructions of the spicules display contrasting layers identical to those in the lenses (Fig. 6), indicating that the layered distribution of Mg-rich nanoparticles is common to the organism’s other calcitic parts as well, and is probably related to the formation mechanism of its skeletal parts. In Fig. 6, the bright layers are denser than the dark layers. Since the Mg-rich nanoparticles are coherent with the matrix, the lattice size does not change significantly between the layers, but there is a change in the elemental composition. Thus, the denser layers contain less Mg i.e., these are the layers that are depleted of Mg-rich nanoparticles. The less dense layers contain more Mg, i.e., they contain more Mg-rich nanoparticles. Intensity line plots of these reconstruction images reveal an oscillation periodicity of ~1 µm (Supplementary Fig. 4).

### Formation model

Considering the extreme complexity and efficiency of the brittle star’s skeletal nanostructure, it is crucial to understand the mechanism of its formation. Some calcitic and aragonitic biominerals (such as mollusk shells^25^ or the sea urchin spicule^26^, spine^27^, and teeth^28^) have been shown to be formed through the crystallization of an amorphous precursor^25–29^. That non-classical crystallization pathway, allows the formation of out-of-equilibrium supersaturated solid solutions (such as high-Mg calcite) as well as the formation of intricate rounded crystal shapes^29^. Since this was also found to be the case with respect to the calcitic components of the brittle star, it is reasonable to assume that those components were formed via a Mg-rich amorphous calcium carbonate (Mg-ACC) precursor. The exact structure and even the state of the amorphous ACC precursor can vary^30–33^; the latter has indeed been shown to exist as a gel^34^ or as a liquid^20,35–41^. Notably, liquid ACC phase has been shown to be stabilized by the presence of acidic proteins, a process called polymer-induced liquid precursor (PILP) process^20,38–41^. It was moreover shown that a much lower amount of protein was necessary to induce the liquid amorphous precursor phase when Mg was present in the system^41^. Likewise, we assume that the amorphous precursor of the brittle star skeletal parts is in a gel or liquid-like state, possibly stabilized by both the Mg and the low amount of proteins present in the system. Below we present a model depicting the formation of the brittle star Mg-calcite nanostructure from an amorphous liquid or gel-like Mg-ACC precursor to a Mg-calcite nanostructure containing periodic layers with varying concentrations of coherent Mg-rich nanoinclusions. In this scenario, the initial stage is the liquid or gel-like supersaturated Mg-ACC precursor formed as a result of vital activity of the brittle star. Formation of the nanostructured Mg-calcite then occurs in two steps. First, the Mg-ACC precursor undergoes a spinodal decomposition into Mg-rich nanodomains and a Mg-depleted ACC matrix. In the second step, this amorphous reorganized precursor crystallizes from a single nucleation point and through an advancing crystallization front that creates the layered structure of the Mg-rich nanodomains by the routes considered below.

### First step: spinodal decomposition of Mg-ACC precursor

Thermodynamic studies have shown that Mg-calcite can be considered as similar to a binary solid solution of its two end-members, calcite, and dolomite^42,43^. According to this approach, the free energy of mixing of Mg-calcite can be written using the two-parameter representation of the Gibbs free energy of mixing:^43^

1 $$\begin{array}{l}\Delta G\left( {X_{\mathrm{D}}} \right) = RT\left( {X_{\mathrm{C}}\ln X_{\mathrm{C}} + X_{\mathrm{D}}\ln X_{\mathrm{D}}} \right) + X_{\mathrm{D}}\left( {1 - X_{\mathrm{D}}} \right)\\ \left[ {A_0 + A_1\left( {2X_{\mathrm{D}} - 1} \right)} \right],\end{array}$$

where *X*_C_ is the mole fraction of calcite and *X*_D_ is the mole fraction of dolomite, *X*_C_ = (1 − *X*_D_), and *R* is the gas constant. According to Busenberg and Plummer^42^, *A*_0_ = 12.6 ± 0.2 kJ.mol^−1^ and *A*_1_ = 4.7 ± 0.1 kJ.mol^−1^, as fitted from their experimental solubility results. The similar values of *A*_0_ = 12.4 kJ.mol^−1^ and *A*_1_ = 2.5 kJ.mol^−1^ were estimated by Lerman^43^ by extrapolating *A*_0_ and *A*_1_ values from high-temperature experimental compositions of Mg-calcite phases^44^. Spinodal decomposition of this solution occurs if the second derivative of the free energy is negative:

2 $$\frac{{\partial ^2G}}{{\partial X_{\mathrm{D}}^2}} = \frac{{RT}}{{X_{\mathrm{D}}\left( {1 - X_{\mathrm{D}}} \right)}} - 2A_0 - 6A_1\left( {2X_{\mathrm{D}} - 1} \right) \, < \, 0.$$

At room temperature and using the values of *A*_0_ and *A*_1_ constants from Busenberg and Plummer^42^, this condition is satisfied for 0.14 < *X*_Mg_ < 0.48 (*X*_Mg_ *= X*_D_/2) (Supplementary Fig. 5). The occurrence of spinodal decomposition is conditional on the diffusion of Mg and Ca, which is too slow in solid magnesian calcites under ambient conditions but can be fast enough in liquids and gels. Here, we assumed that Mg-ACC solutions are gel or liquid-like and are thermodynamically similar to the corresponding crystalline solution. The presence of charged organic molecules in a Mg-ACC precursor may alter thermodynamic and kinetic parameters of the system. However, we believe that such low amounts of proteins as observed in brittle star influence mainly the kinetic parameters such as effective diffusion coefficients of Mg and Ca ions. The supersaturated Mg-ACC could then be approximated by a similar binary solution with two end-members, amorphous CaCO_3_ and amorphous Ca_0.5_Mg_0.5_CO_3_, which—like magnesian calcite—can be unstable. Therefore, spinodal decomposition should occur for 0.14 < *X*_Mg_ < 0.48, similarly to crystalline magnesium calcite. In contrast to solid magnesium calcites, spinodal decomposition of liquid or gel-like Mg-ACC is kinetically possible at room temperatures (see Supplementary Note 1). Moreover, we can expect that the Mg-ACC precursor of brittle star lenses contains the same global amount of Mg as the lenses, being of *η* = 15.2 mol% (with *η* = Mg/(Ca + Mg)), as obtained by Polishchuk et al.^6^ Therefore, at this Mg concentration, the above thermodynamic and kinetic considerations predict a spinodal decomposition of the gel-like Mg-ACC precursor. Thus, the hypothesis of spinodal decomposition of the Mg-ACC precursor to Mg-depleted calcite matrix and Mg-rich nanodomains is reasonable. We emphasize that the spinodal decomposition is indeed the most probable way to obtain numerous nanoparticles that are homogeneously distributed throughout the matrix. Other possibilities such as multiple nucleation of Mg-rich amorphous or crystalline particles from a metastable amorphous state are less likely from the kinetic point of view due to comparatively slow nucleation rates.

**Fig. 7:**
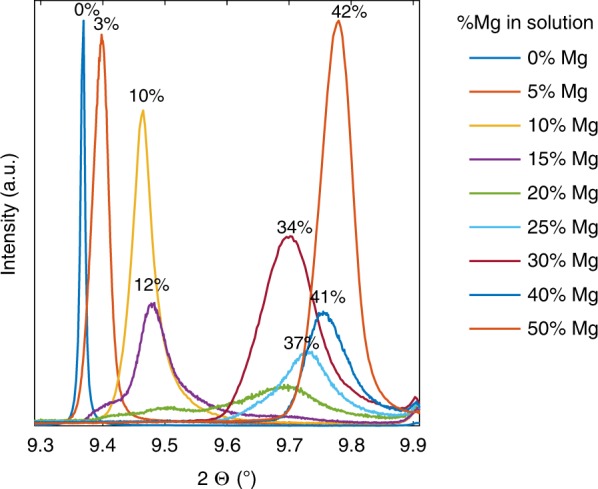
Instability of Mg-CaCO_3_ for a range of Mg composition. (104) XRD peak from Mg-calcite crystallized from hydrothermally treated Mg-ACC in solution. Data were acquired by synchrotron radiation (λ = 0.496 $\dot{A}$). Numbers near the peaks are the η (Mg/Ca + Mg) in the calcite crystals, obtained from the refined lattice parameters and the equations linking the lattice parameters and η obtained by Zolotoyabko et al.^24^. The diffractograms are normalized to the highest diffraction peaks (calcite (104) peak for the powders precipitated from solutions containing 0 or 50% Mg in solutions, and aragonite peak for the other powders)

To support the hypothesis that the homogeneous 15-mol% Mg-ACC is unstable by analogy with the 15-mol% Mg-calcite, we synthesized Mg-ACC by fast mixing of solutions of CaCl_2_ and MgCl_2_ with solutions of Na_2_CO_3_. The Mg-ACC in solution can be analogous to a gel-like phase. We then crystallized the Mg-ACC solutions by exposing them to mild hydrothermal conditions (135 °C, 3 bar). This synthesis protocol is one of the protocols in which Mg-ACC is used as a precursor to the subsequently crystallized high-Mg calcite phases^45,46^. The outcome of the hydrothermal treatment is the fast crystallization of the Mg-ACC, which otherwise, in its amorphous state, is stable over time^47^. In this way, we synthesized Mg-calcite powders from a series of Mg-ACC precipitates, which we had obtained using different concentrations of Mg in solution (see Experimental section). XRD of the synthesized powders indicated mixtures of Mg-calcite and aragonite (see Supplementary Table 3 for the relative amounts of aragonite). The amount of Mg in the synthesized calcite can be visualized by the shift of the calcite phase in the diffractogram (Fig. 7), and can be quantified from the calcite lattice parameter values, which decrease with the amount of Mg present in the calcite phase^24^. From the XRD data it appears that intermediate Mg-calcites (0.10 < *η* < 0.34) do not form; instead, the precipitates obtained are a mixture of higher and lower Mg-calcite phases and a large amount of aragonite. The absence of mid-Mg calcite (0.10 < *η* < 0.34) when crystallized at moderately high temperatures indicates that a decomposition might have already occurred in the amorphous precursor. Indeed, since the diffusion is limited in the solid phase (even at 135 °C), the decomposition probably already occurs in the gel-like amorphous state rather than in the crystalline phase, thus supporting our assumptions.

We would like to note that the synthesis of high-Mg calcite is not trivial. Indeed, at ambient conditions, crystallization of Mg-ACC does not form high-Mg calcite and aragonite is favored, unless some specific organic molecules are involved in the synthesis^41,48^. Here, we have established that an extremely low amount of proteins is present in the biomineral. Moreover, the above theoretical considerations rely on established models that do not involve organic matter. Therefore, although some biological control involving organic molecules certainly takes place, we believe that Mg is the key player in the Mg-ACC spinodal decomposition process. To prove this point, we have chosen a protocol that isolates the effect of Mg amount, in the absence of organic additives (even though this protocol does not reproduce the biological ambient conditions of the biomineral formation). To further evaluate the effect of organic molecules on the behavior of Mg-ACC, we performed identical Mg-calcite syntheses, but in the presence of aspartic acid, a common amino acid involved in biomineralization. In these conditions, the intermediate-Mg calcite (0.10 < *η* < 0.34) could form (Supplementary Fig. 6), revealing that these otherwise unstable Mg-ACC compositions can be stabilized by the presence of organic molecules. Additional syntheses, in the presence of poly-aspartic acid, a polypeptide used to model the proteins involved in biomineralization^38–41^, reconfirmed this result (Supplementary Fig. 7). Therefore, we suggest that both the amount of Mg corresponding to Mg-ACC instability range and the low amount of protein involved are specificities of the brittle star skeletal parts that enable spinodal decomposition of the Mg-ACC precursor, to Mg-rich domains and Mg-depleted matrix. This biomineral formation mechanism therefore uses the thermodynamic conditions created by its biological environment but this formation step is not directly biologically controlled; it can rather be explained by classical thermodynamics, a phenomenon that was shown in another biomineral, the prismatic layer P. Nobilis^49,50^.

### Second step: crystallization and layered structure formation

The next step in the formation sequence is the crystallization of the decomposed Mg-ACC precursor.

**Fig. 8:**
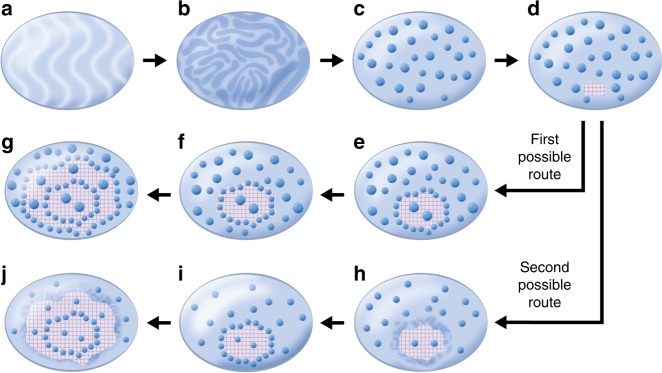
Proposed mechanism of formation of layered structure. **a** Gel-like or liquid-like Mg-ACC precursor containing 15% MgCO_3_. **b** Decomposition starts from growing fluctuations in concentration (spinodal decomposition of Mg-ACC solution). **c** Large Mg-concentration fluctuations result in formation of Mg-rich nanoparticles. **d** At some point, a nucleus of crystalline Mg-depleted calcite appears. **e,** **f** First possible crystallization route: **e** the crystalline nucleus grows through the gel, rejecting small particles (below a critical size) and incorporates large particles (above the critical size). **f** Local gel viscosity increases owing to the increase in volume fraction of the particles reaching the critical value at which particles are trapped and coherently crystallize. **g** The process is repeated. **h**–**j** Second possible crystallization route: **h** Mg exclusion from crystallizing calcite and Mg enrichment of the vicinal Mg-ACC leads to secondary spinodal decomposition near the crystallization front. **i** Resulting formation of additional Mg-rich nanoparticles. **j** Advancement of crystallization front leading to formation of particle-enriched crystalline layer; thereafter the process of Mg exclusion and Mg-ACC secondary spinodal decomposition is repeated

The crystallization probably starts in the Mg-depleted matrix, since Mg-rich ACC is more stable against crystallization (Fig. 8d)^47,51^. We consider two possible crystallization routes leading to the formation of the layered structure (Fig. 8e–j). One possible route is depicted in Fig. 8e–g: The crystallization expands through an advancing crystallization front that faces a matrix containing high-Mg nanodomains, a scenario that can be comparable to the extensively studied case^52–55^ in which particles face a solidification front, on a larger length scale, such as in the case of freezing water-saturated soil^53^. Following the commonly accepted model for the latter situation, we can assume that the crystallization front and the particles are subjected to attractive and repulsive forces, which alternatively exceed one another, causing alternate embedding and repelling of the particles by the crystallization front. The disjoining force responsible for this repelling is still a matter of debate; it is usually assumed to result from van der Waals and electrostatic interactions between the dispersed nanoparticles and the solidification front. Here, we assume that a Columbian repulsive force exists between them, *F*_q_, maybe as a result of absorption of negatively charged organic and water molecules, both on the nanodomains and the crystallizing surface (the presence of these molecules at the crystallization front can result from organic molecules exclusion during crystallization^20^). The disjoining force is countered by an attractive force, the lubrication force (*F*_*μ*_) that is associated with the flow of viscous fluid near the base of the dragged particle when repelled by the crystallization front^54–56^. This lubrication force depends on the viscosity of the suspension medium facing the crystallization front (*μ*—itself depending on the volume fraction of the particles *ϕ*^56^) and on the size of the particles: *F*_μ_(*R*, *μ*(*ϕ*)). The transition from rejection to encapsulation of the particles is achieved when *F*_q_ = *F*_*μ*_. This condition allows us to derive the maximum radius of rejected particles *R*_m_(*μ*). The larger particles (*R* > *R*_m_(*μ*)) are trapped by the crystalline front and encapsulated (or coherently crystallized in our case), while smaller particles (*R* < *R*_m_(*μ*)) are repelled and pushed to the gel. Our estimations yield a critical radius of repelled particles *R*_m_(*μ*_0_) = 5 nm (see Supplementary Note 2). Polishchuk et al. assessed the average diameter of the nanoparticles as ∼5 nm (meaning $\bar r \sim 2.5{\mathrm{nm}}$)^6^; therefore, almost all of the particles should be rejected by the crystallization front (*R* < *R*_m_(*μ*_0_)). The crystallization front then sweeps up the Mg-rich nanodomains while expanding radially (Fig. 8e). However, if the crystallization front repels a relatively high amount of nanoparticles, the volume fraction of the nanoparticles in proximity to the crystallization front will increase. The viscosity of the gel facing the crystallization front will then reach a critical value above which the lubrication force exceeds the repulsion force; the nanodomains will then not be repelled into the gel but will rather be pushed towards the crystallization front and forced to crystallize within the calcite matrix (Fig. 8f), creating a nanoparticle-rich crystalline layer. After incorporating the accumulated nanoparticles in the crystalline phase, the crystallization front will again face the lowered viscosity gel (with a low concentration of Mg-rich nanodomains). The process is then repeated, creating an alternating nanoparticle-rich/nanoparticle-poor layered structure (Fig. 8e–g).

An alternative or synergetic route leading to the oscillatory distribution of Mg-rich nanoparticles is depicted in Fig. 8h-j: This route relies on the phenomenon of impurity exclusion during ACC crystallization. ACC crystallization in the presence of polypeptides was indeed shown to form “transition bars”, formed as a result of exclusion of the polymer impurity during liquid ACC crystallization^20^. Similarly, the crystallization of Mg-depleted ACC matrix can be accompanied by the exclusion of Mg ions into a diffusion zone adjacent to the crystallization front. Thus, the crystallization front can be considered as a source of Mg, and the adjacent Mg-ACC diffusion zone becomes enriched in Mg. Due to this enrichment, the Mg content in the vicinity of the crystallization front can exceed the critical Mg content above which the Mg-ACC matrix becomes unstable against spinodal decomposition (Fig. 8h). A new secondary spinodal decomposition will then take place and result in the formation of new Mg-rich nanodomains. As a result, the density of Mg-rich nanodomains will be higher near the crystallization front (Fig. 8i). The advancement of crystallization front will then form a new crystalline layer with a higher concentration of Mg-rich nanodomains. This process is repeated, (Fig. 8j) creating the layered structure (see details in Supplementary Note 3).

## Discussion

Previous studies of the *O. wendtii* brittle star lenses have revealed their excellent optical and mechanical properties as well as their elaborate nanostructure^5,6^, but how they form is largely unknown. In this study we observe the widespread finding that the coherent Mg-rich nanoparticles previously discovered in the lenses^6^ also exist in other calcitic parts of this organism, namely the spicules, the teeth and the arm vertebrae. The Mg-rich nanoparticles are observed by using complementary X-ray and electron methods that proved that the Mg-rich particles are arranged in alternating nanoparticle-rich and nanoparticle-poor layers. Based on previous studies from the literature as well as experimental data on the newly discovered nanostructure of the brittle star’s calcitic parts, we developed a scenario of its formation from a supersaturated gel or liquid-like Mg-ACC precursor. An important aspect of this scenario involves the instability of Mg-ACC with 0.14 < *η* < 0.48 (*η* = Mg/(Ca + Mg)), leading to spinodal decomposition of the 15%-Mg-ACC precursor into Mg-rich nanodomains and a Mg-depleted matrix. Crystallization of this decomposed precursor from a single nucleation point advances via the crystallization front through a suspension of Mg-rich ACC, which results in the formation of single-crystal Mg-calcite with coherently embedded Mg-rich calcite nanoparticles distributed in alternating particle-enriched and particle-depleted layers. The spinodal decomposition within Mg-ACC has been supported by experimental findings in a synthetic assay. We also show that organics can alter the kinetics and suppress the spinodal decomposition. The finding that the mineralized parts of the brittle star comprise very low amounts of organics, as compared to that found in other organisms, suggests that this is a key parameter in the coherent nanoprecipitates formation via spinodal decomposition and may explain why this phenomenon was not observed to date in other organisms. The Mg-ACC instability discovered here is of fundamental importance, owing to the preponderance of Mg-ACC in biominerals or as a precursor to biominerals. In future studies, proposed Mg-ACC stability model should be tested on different biominerals or synthetic systems.

## Methods

### *O. wendtii* collection and bleaching

Samples of *O. wendtii* were kindly provided by Dr. Gordon Hendler, Curator of the Echinoderm Department at the Natural History Museum of Los Angeles. The samples were collected in Belize during the 1980s and 1990s and preserved in AR grade ethanol. The arms of the brittle star were cut with a scalpel into segments and bleached in a solution of deionized (DI) water and sodium hypochlorite, with a 2:1 volume ratio of NaOCl:DI for 8 h. To avoid dissolution of calcite in the liquid we added 2% (20 g.L^−1^) sodium carbonate Na_2_CO_3_ to the DI water. After removal of the organics the solution was poured out, and the mineralized phase was rinsed several times with DI water and left to dry in the air.

### AAA

Powdered sample was fully dissolved in 200 µL of a 1 M HCl solution. DI water was added to complete 10 mL total. After hydrolysis, the sample was left to dry and then dissolved in DI water, a few µL were then taken to be measured and analyzed for amino acid.

### HRSEM

Carbon coated samples were imaged with the Zeiss Ultra-Plus FEG-SEM, using accelerating voltages of 4–20 kV.

### HRTEM

The FEI Titan 80–300 FEG-S/TEM system was used on FIB-cut cross-sections, in bright-field TEM mode. The FEI Titan Cubed Themis G2 60–300 was used on a FIB-cut cross-section, in HAADF-STEM mode, at extremely low current of 7.5 pA in order not to damage the samples.

### Synchrotron HRXRD

Diffraction patterns of bleached powdered samples were acquired in the ID-22 beamline at the European Synchrotron Radiation Facility (ESRF), at wavelength of 0.399 Å and 0.496 Å. The diffraction patterns were analyzed by Rietveld refinements using GSAS II software^57^.

### Nano-HT

Nano-tomography datasets were obtained at the ID-16B beamline of the ESRF. Bleached samples of dorsal arm plates (DAPs) were sectioned with a scalpel, and small fragments containing several lenses were mounted on the tip of a metal needle and fixed with epoxy. To isolate a single lens, the small DAP fragment was fixed onto an automatic stage equipped with a CO_2_ laser that cut the superfluous parts in order to isolate one lens. The samples were positioned at four different distances between the focal plane of the X-ray beam, which focused down to a spot size of 50 × 50 nm^2^ using two multilayer-coated Si mirrors in Kirkpatrick-Baez (KB) configuration operated under pink beam mode at 29.6 KeV and 17.5 KeV, and the camera. While the sample rotated (360°), 2001 high-resolution images were collected. The datasets were then first processed using a phase retrieval algorithm based on ESRF in-house software using the GNU Octave programming environment^58^. In a second step, 3D volumes using the filtered phase maps were reconstructed using back projection with the ESRF python software PyHST2^59^.

### XRF tomography

XRF tomography was also performed at the ID-16B beamline of the ESRF, with the same focusing optics. In this set-up, the sample was mounted on a rotating stage and horizontal line-scans were performed using the focused beam for each rotation interval until a 360° rotation is complete. Measurements were performed at 17.5 KeV. The XRF-emitted signal was collected by detectors situated perpendicular to the incident beam, then fitted and processed using the Python Multi-Channel Analyzer (PyMCA) program^60^. Elemental sinograms from all detectors were combined and were then further reconstructed by use of the XRDUA software^61^ that uses an iterative algorithm. The obtained 2D elemental distribution slices were finally partially corrected for self absorption.

### FIB

We used the FEI Helios NanoLab G3 UC Dual Beam FIB in our experiment. Ion-milling was performed utilizing high gallium milling currents and accelerating voltage for 1 min 40 s at 0.23 nA. These conditions allowed ~400 nm milling depth, as measured from close-ups images of the cross-section of the milled box. Samples were embedded in an epoxy resin and polished using a Struers Rotopol-31 + RotoForce-4 polishing machine and successively fine diamond lapping films.

### Mg-calcite syntheses

Solutions of 0.2 M Na_2_CO_3_ and solutions of 0.2 M CaCl_2_ and MgCl_2_ (with varying relative amount of MgCl_2_) were mixed, forming colloidal solutions of Mg-ACC in water. In some of the syntheses, L-aspartic acid (0.1 M) and poly-aspartic acid (200 µg.mL^−1^, M.W. = 2000–11,000) were added to the Na_2_CO_3_ solutions. The beakers were then placed in an autoclave, at 135 °C, 3 bar, for 2 h. The solutions were filtered, rinsed, and dried.

## Supplementary information

Supplementary Information

## Data Availability

The data that support the findings of the study are available from the corresponding author upon reasonable request.
