# Supplementary material for: From spinodal decomposition to alternating layered structure within single crystals of biogenic magnesium calcite
Source: Nat Commun. 2019 Oct 8;10:4559. doi: 10.1038/s41467-019-12168-8 (PMC6783414; doi:10.1038/s41467-019-12168-8)
Supplement: Supplementary file 1 — Supplementary Information [file 41467_2019_12168_MOESM1_ESM.pdf]

Supplementary Information

From spinodal decomposition to alternating layered structure within single crystals of biogenic magnesium calcite

Seknazi et al.

Supplementary Figures

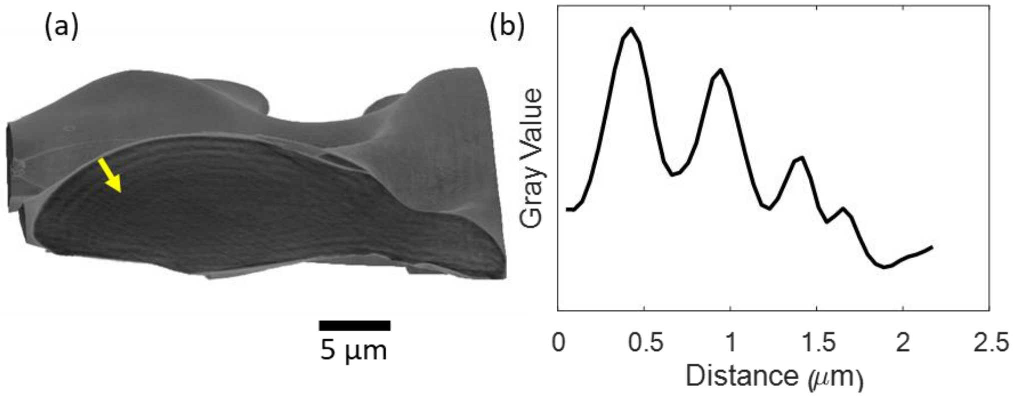

Supplementary Figure 1*. Nano-HT reconstruction of a sample that was heated at 300°C, for 70 min, showing density oscillations perpendicular to the sample surface (pixel size is 35 nm). b) Intensity plot from the yellow line*

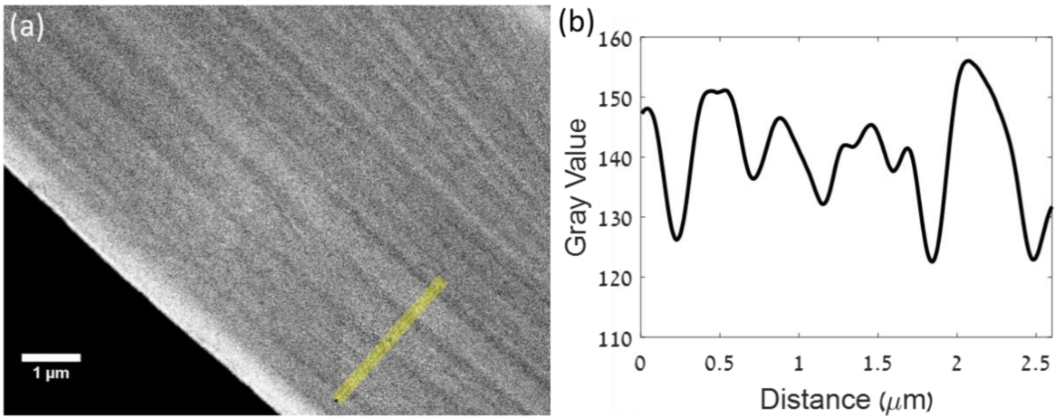

Supplementary Figure 2*. a) BSE image of a lens embedded in epoxy and polished. b) Intensity plot from the yellow line*

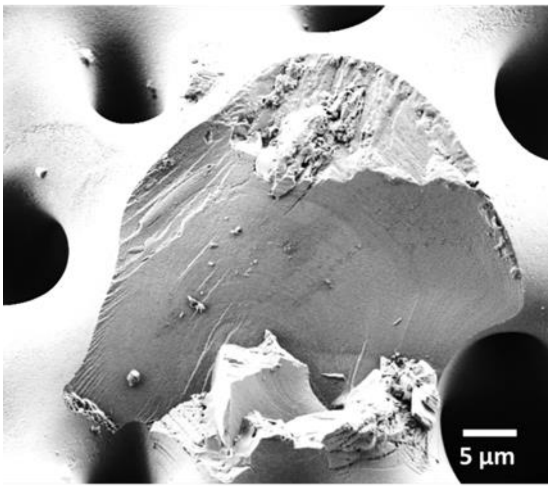

Supplementary Figure 3*. HRSEM (SE) image from a broken lens.*

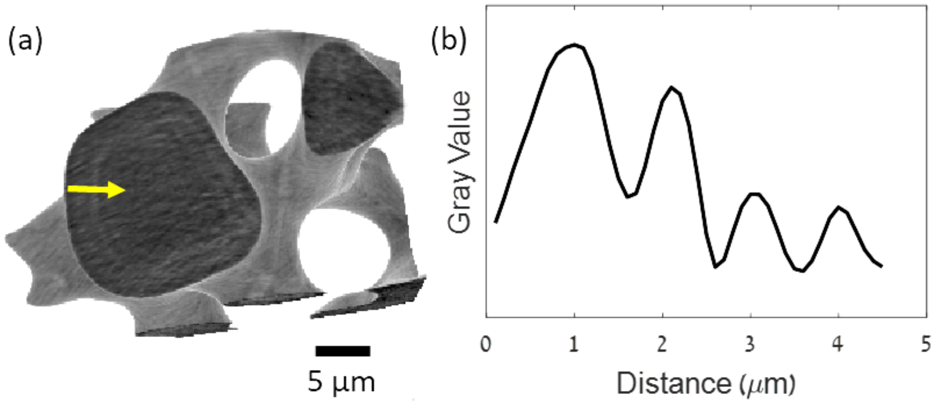

Supplementary Figure 4. *a) Nano-HT reconstruction of a spicule sample at room temperature, showing density oscillations perpendicular to the sample surface (pixel size is 35 nm). b) Intensity plot from the yellow line.*

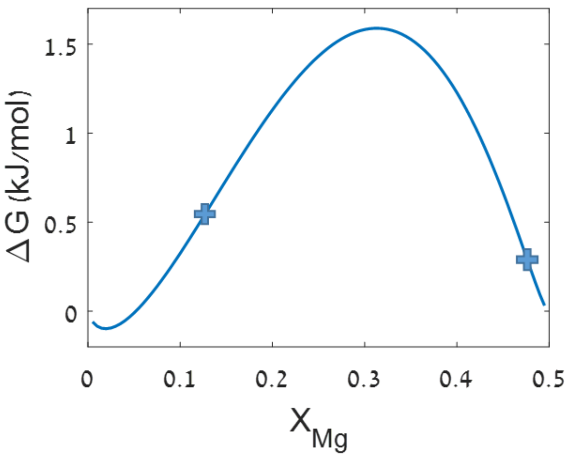

Supplementary Figure 5. *Free energy of Mg-calcite taken as a binary solid solution of calcite and dolomite at T = 298K with the constants A_0 and A_1 from Busenberg and Plummer.*[1] *Crosses correspond to zero second derivative of the free energy (X_{Mg} = 0.14 and X_{Mg} = 0.48).The region of negative second derivative of the free energy is in between the crosses.*

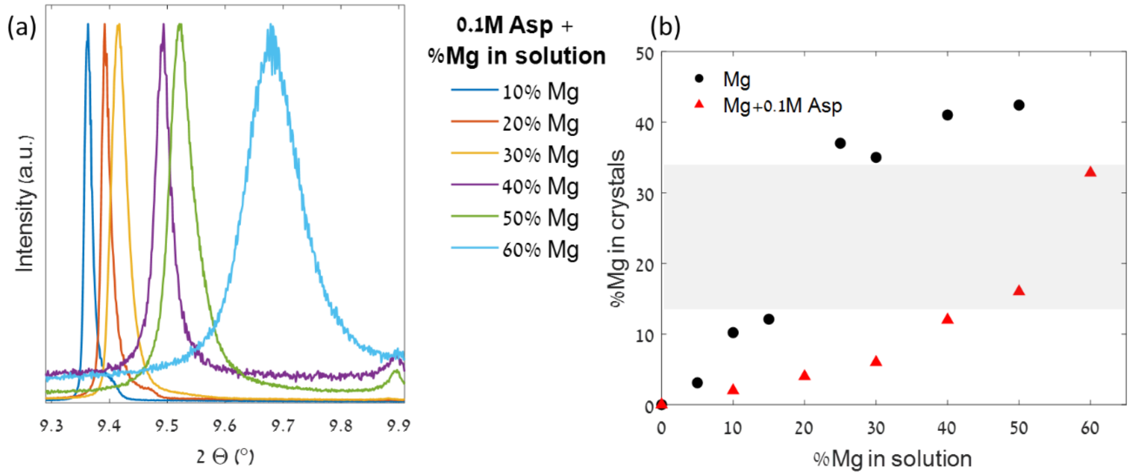

***Supplementary Figure 6**. (a). (104) XRD peak from Mg-calcite crystallized from hydrothermally treated Mg-ACC in solution in the presence of 0.1M Aspartic acid. Data were acquired by synchrotron radiation (λ=0.496 Å). (b) (Mg/Ca+Mg) in the calcite crystals, obtained from the refined lattice parameters and the equations linking the lattice parameters and the amount of Mg in the crystal obtained by Zolotoyabko et al.,[2] as a function of amount of Mg in solution, in the absence (lack circles) and presence (red triangles) of aspartic acid. The grayed area represents the range for which Mg-calcite did not form.*

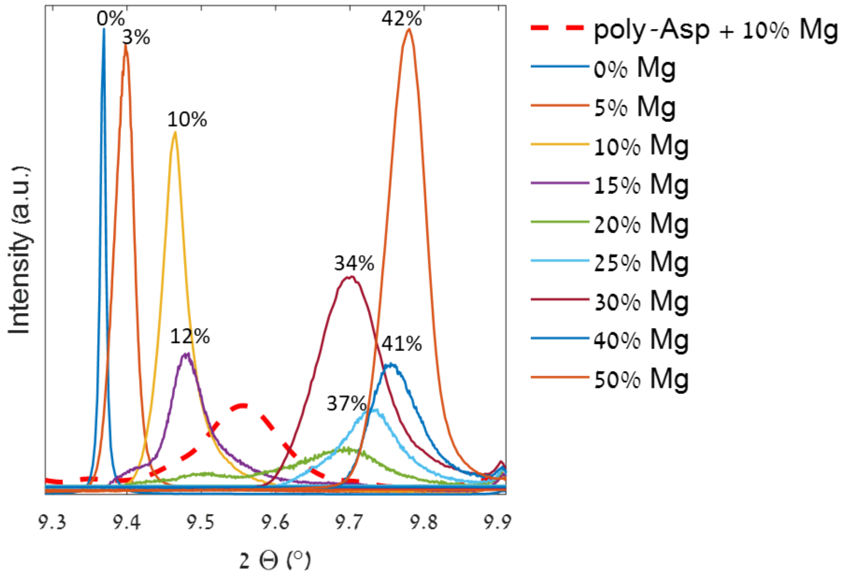

***Supplementary Figure 7**. Data from Figure 7 (in manuscript), plotted together with the (104) XRD peak from Mg-calcite crystallized in an identical manner but in the presence of polyaspartic acid (200 μg.mL^{-1} polyaspartic acid and 10%Mg in solution). Numbers near the peaks are the η (Mg/Ca+Mg) in the calcite crystals, obtained from the refined lattice parameters. XRD from the sample synthesized in the presence of polyaspartic acid was acquired with a CuKα radiation (λ=1.5406 Å, rescaled at λ=0.496 Å), the other XRD data were acquired by synchrotron radiation (λ=0.496 Å).*

Supplementary Tables

AA	weight%
asp	0,000202
ser	0,000131
glu	0,000252
gly	0,000193
ala	0,000122
val	0,000109
Ile	7,95E-05
leu	0,000154
phe	7,74E-05
Total	0,00132

Supplementary Table 1. *Amino Acid Analysis results*

	Temperature	phase	wt%	a-parameter Å	c-parameter Å	wR %	GOF
spicules	RT	low-Mg calcite	100	4.92857(3)	16.77874(7)	14.4	4.9
	RT after 400°C	low-Mg calcite	97.7	4.9338(2)	16.8066(5)	11.8	4.6
		high-Mg calcite	2.3	4.827(5)	16.19(1)		
arm vertebrae	RT	low-Mg calcite	100	4.92680(2)	16.776904(6)	14.0	3.2
	RT after 400°C	low-Mg calcite	97.7	4.9326(1)	16.8015(3)	12.5	2.8
		high-Mg calcite	2.3	4.836(2)	16.156(5)		
teeth	RT	low-Mg calcite	100	4.92827(2)	16.77693(5)	16.5	3.6
	RT after 400°C	low-Mg calcite	94.5	4.9353(2)	16.8159(4)	12.3	2.5
		high-Mg calcite	5.5	4.837(2)	16.163(4)		

Supplementary Table 2. *Rietveld refinement results: Lattice parameters, phase fraction and goodness of fit parameters.*

%Mg in solution	Wt% aragonite
0	48
5	67
10	69
15	77
20	81
25	79
30	65
40	78
50	25

Supplementary Table 3. *Relative amount of aragonite (wt%) obtained during the synthesis of Mg-calcite, extracted from Rietveld refinement results.*

Supplementary Notes

Supplementary Note 1: Kinetics of the spinodal decomposition of gel-like Mg-ACC

Consider a homogeneous, quasi-binary liquid solution L(A-B) thermally treated at a temperature T below the critical point, thereby providing spinodal decomposition. The equation describing evolution of the concentrations of element A and element B over time in the liquid (or gel) matrix M ($C_A+C_B=1$) can be written in the form specified in [3]. Assuming that we can neglect non-linear concentration derivatives, these equations can be written in the following one-dimensional form:

$$\frac{\partial C_{\mathrm{A}}}{\partial t} = M_{\mathrm{AM}}\left[G_{\mathrm{AA}} \cdot \frac{\partial^2 C_{\mathrm{A}}}{\partial x^2} - 2\kappa_{\mathrm{AB}}\frac{\partial^4 C_{\mathrm{A}}}{\partial x^4}\right], \tag{1}$$

where $G_{\mathrm{AA}} \equiv \frac{\partial^2 G}{\partial C_{\mathrm{A}}{}^2}$, G is the molar Gibbs free energy and M_{AM} is the effective mobility of element A in the gel matrix M. The gradient energy coefficient κ_{AB} can be evaluated according to the known relation for solid binary alloys and corrected for gel solutions:

$$\kappa_{\mathrm{AB}} = \frac{2\alpha}{3} a^2 \Delta h_{0.5}^{\mathrm{AB}}, \tag{2}$$

where $\Delta h_{0.5}^{\mathrm{ij}}$ is the mixing enthalpy of the corresponding equimolar $i{-}j$ binary alloy, a is the interatomic distance, and α is a gel correction parameter.

Possible fluctuations in concentration of element A that satisfy Supplementary Equation 1 can be written in the form:

$$\Delta C_{\mathrm{A}} = A_0 e^{Qt} \cos(\beta x), \tag{3}$$

where $Q = -M_{\mathrm{AM}}\beta^2(G_{\mathrm{AA}} + 2\kappa_{\mathrm{AB}}\beta^2)$. The fluctuation in concentration will grow for $Q > 0$, i.e. $G_{\mathrm{AA}} < -2\kappa_{\mathrm{AB}}\beta^2$. The maximum rate of fluctuation growth corresponds to $\beta_{\mathrm{m}}^2 = -G_{\mathrm{AA}}/4\kappa_{\mathrm{AB}}$:

$$Q_{\mathrm{m}} = M_{\mathrm{AM}}\frac{(G_{\mathrm{AA}})^2}{8\kappa_{\mathrm{AB}}}. \tag{4}$$

Let us now estimate possible values of this amplification factor and the wavelength of fluctuations $\lambda_{\mathrm{m}} = 2\pi/\beta_{\mathrm{m}}$ for the case of the Mg-ACC aqueous matrix. Using Equations 1 and 2 for the Gibbs free energy,

and Supplementary Equation 2, we can write $\lambda_{\mathrm{m}} = 2\pi a\sqrt{-2\alpha A_0/3G_{\mathrm{AA}}}$. For T = 300K and concentrations slightly above the critical concentration X_c = 0.14, the second derivative can be about G_{AA} = –(0.006÷0.4) kJ.mol^{-1}, and $\lambda_{\mathrm{m}} = (4 \div 34)\sqrt{\alpha}$ nm. The value of the amplification factor can be estimated using the mobility $M_{\mathrm{AM}} = (V_m/RT)\bar{D} \approx 1.5 \cdot 10^{-23} m^5 (Js)^{-1}$, where $\bar{D} \approx 10^{-15}$, $m^2.s^{-1}$ is the diffusion coefficient in the gel, and V_{m} is the molar volume of calcite. For applied values of G_{AA} and κ_{AB} it can be seen that Q_{m} varies over a wide range, from ~$(0.02/\alpha)$ s^{-1} for $G_{\mathrm{AA}} = -0.006$ kJ.mol^{-1} to $(90/\alpha)$ s^{-1} for $G_{\mathrm{AA}} = -0.4$ kJ.mol^{-1}. Assuming α = 10÷100, we can estimate the time needed to increase the fluctuation for 5 orders of value $t_5 = (5/Q_{\mathrm{m}})\, ln\, 1\, 0$ as $5.8 \cdot (10^3 \div 10^4)$ s for $G_{\mathrm{AA}} = -0.006$ kJ.mol^{-1}, and $(1.3 \div 13)$ s for $G_{\mathrm{AA}} = -0.4$ kJ.mol^{-1}. We can conclude here that the spinodal decomposition becomes increasingly faster with growth of the average Mg concentration above the critical value.

Supplementary Note 2: The layered structure formation, 1st route: alternating repelling and attachment of the Mg-rich particles

We assume that the Mg-rich nanoparticles do not become attached to the growing crystalline front because of some Columbian repulsive force caused by absorbed negatively charged organic and water molecules, both on the nanodomains and the crystallizing surface. Assuming approximately homogeneous distribution of charged molecules throughout the crystallizing surface, the corresponding disjoining force can be written as follows:

$$F_{\mathrm{q}} = \frac{\sigma q_{\mathrm{p}}}{2\varepsilon_0} \quad . \qquad (5)$$

where σ is the surface charge density, q_{p} is the charge accumulated by a nanodomain, ε_0=8.85·10^{-12} F.m^{-1} is the electric constant.

The crystallization front expands at a rate V and drags the Mg-rich nanoparticles. The balancing force in a viscous media was derived as a lubrication force caused by viscous fluid flow near the base of

particle.[4–7] In the case of small spherical particles of radius R near a flat interface, the viscous drag force is: [4–7]

$$F_\mu = 6\pi\mu V\frac{R^2}{d} \tag{6}$$

where μ is the suspension viscosity. The particles are rejected by the expanding crystalline front if the disjoining force exceeds the viscous lubrication force. The transition from rejection to encapsulation of the particle occurs when:

$$F_q = F_\mu \text{ i.e. } \quad 6\pi\mu V\frac{R^2}{d} = \frac{\sigma q_\mathrm{p}}{2\varepsilon_0}. \tag{7}$$

This condition allows to derive the maximum radius of rejected particles, $R_\mathrm{m}(\mu)$:

$$R_\mathrm{m}(\mu) = (\frac{\sigma q_\mathrm{p} d}{12\pi\mu V\varepsilon_0})^{1/2} \tag{8}$$

The larger particles ($R > R_\mathrm{m}(\mu)$) are trapped by the crystalline front and encapsulated (or coherently crystallized in our case), while smaller particles ($R < R_\mathrm{m}(\mu)$) are repelled and pushed to the gel.

According to theory of Vand,[8] the viscosity of a gel containing a volume fraction of suspended particles φ can be written as:

$$\mu(\varphi) = \frac{\mu_0}{(1-\varphi-q\varphi^2)^k} \tag{9}$$

where k = 2.5 for spherical particles and k > 2.5 for elongated particles, and q~1 is a fitting parameter. Using the reasonable values of d = 0.5 nm, $\mu_0 = 16 Pa\cdot s$, V = 0.01 µm.s^{-1}, $\sigma = 10^{12}\bar{e}$ m^{-2}, $q_\mathrm{p} = \bar{e}$, where $\bar{e}$ is elementary charge, as well as an initial volume fraction $\varphi_0 \approx 0.08$ (as estimated by Polishchuk et al.[9]), yields a critical radius of repelled particles $R_\mathrm{m}(\mu_0) = 5\ nm$. Polishchuk et al. assessed the average diameter of the nanoparticles as ~5 nm (meaning $\bar{r} \sim 2.5nm$)[9], therefore, almost all of the particles should be rejected by the crystallization front. An increase in the effective viscosity results in a corresponding decrease of the critical radius, Supplementary Equation 8. We estimate that the viscosity should increase by a factor of 3 to 4 (see below). If, for example, the viscosity increases by 4-

fold, the critical radius will decrease from 5 nm to 2.5 nm, and almost all particles will then become attached to the crystallization front and crystallize together with the matrix.

Let us consider a crystallization front advancing radially from radius R_1 to radius R_2, and sweeping up all colloidal particles in its path. If N_0 particles were initially distributed in a spherical layer between radii R_3 and R_1 ($R_3 > R_2 > R_1$), with a volume fraction of $\varphi_0 = N_0\bar{r}^3/(R_3^3 - R_1^3)$ ($\bar{r}$ being the average radius of the particles), these particles, after being swept up, are concentrated in the spherical layer between R_3 and R_2, and their volume fraction reaches the value $\varphi_s = N_0\bar{r}^3/(R_3^3 - R_2^3)$. The ratio of particle-depleted to particle-enriched layers can be estimated as $\frac{\Delta L_{12}}{\Delta L_{23}} = \frac{(R_2-R_1)}{(R_3-R_2)} \approx \left(\frac{\varphi_s}{\varphi_0} - 1\right)$. The distance between particles ($d = \bar{r}(4\pi/3\varphi)^{1/3}$) decreases and may reach a critical value ($2\bar{r}$) at $\varphi_s = \pi/6$, when particles meet and agglomerate. In this case, $\frac{\Delta L_{12}}{\Delta L_{23}} \approx \left(\frac{\pi}{6\varphi_0} - 1\right)$. If $\varphi_0 = 0.08$ (as estimated by Polishchuk et al.[9]), the ratio of layer thicknesses should be ~5.5. From the results presented above, this ratio can be estimated as ~(2÷3), which corresponds to $\varphi_s/\varphi_0 \approx (3 \div 4)$, and $\varphi_s = (0.24 \div 0.32)$. For $\bar{r}$ =5 nm, $d_0 \approx$ 18.7 nm and d_s = (13.0÷11.8) nm, this means that the real distance between particle surfaces decreases from 8.7 to 3 nm for $\varphi_s = 0.24$ and to 1.8 nm for $\varphi_s = 0.32$. At such distances, the particles may interact substantially with each other, and by these means form an immobile particle-enriched layer. In other words, when the volume fraction of particles in the gel increases, the effective viscosity of the suspension may also increase substantially, and the particles will be pushed to the crystallization front by the increased viscous force. If, because of the repelled nanoparticles, their local volume fraction increases from the initial value of $\varphi_0 \approx 0.08$ to $\varphi_s = (0.24 \div 0.32)$, the effective viscosity will increase, according to Supplementary Equation 9, by a factor of (2.0 ÷ 3.4).

In order to estimate possible values of particle-depleted and particle-enriched layer widths, one should consider Brownian motion of particles ahead of the crystallization front. Assuming that the swept particles are distributed within the mean squared displacement distance, $\bar{l} = \sqrt{2D_p\tau}$, where $D_p = k_B T/(6\pi\bar{r}\xi)$ is the diffusion coefficient of spherical particles of radius $\bar{r}$ in a viscous liquid ξ, τ is the time needed to reach critical concentration of particles, we can write the ratio of final to initial concentration as following:

$$q \equiv \frac{C_{\mathrm{f}}}{C_{\mathrm{in}}} = \frac{\Delta L_{12}}{\Delta L_{23}} = \frac{V\tau}{\sqrt{2D_p\tau}} \quad (10)$$

where V is the crystallization front velocity. Eliminating τ from this equation, one can find:

$$\Delta L_{12} = \frac{2q^2 D_{\mathrm{p}}}{V} \quad (11)$$

For reasonable values q = (2÷3), $D_{\mathrm{p}} \approx 10^{-15}$ $m^2.s^{-1}$, $V \approx 10^{-8}$ $m.s^{-1}$, the value of ΔL_{12} varies in a wide range (0.8÷1.8) μm, as was observed in our experiments.

Supplementary Note 3: The layered structure formation, 2nd route: secondary Mg-ACC spinodal decomposition

The crystallization of Mg-depleted ACC gel/liquid matrix can be accompanied by the exclusion of redundant Mg atoms and water molecules from the crystallizing calcite to adjacent ACC layer. If the concentration of Mg in the Mg-depleted ACC matrix after spinodal decomposition is C_1^{L}, and in the calcite is $C_1^{\mathrm{S}}, C_1^{\mathrm{S}} < C_1^{\mathrm{L}}$, the crystallization front can be considered as a source of Mg, with approximately constant flux of Mg atoms to the ACC matrix:

$$J_0 = -D\frac{\partial C}{\partial x}\Big|_{\mathrm{cr.front}} = V \cdot (C_1^{\mathrm{L}} - C_1^{\mathrm{S}}) \quad (12)$$

The evolution of Mg concentration in the ACC layer ahead of the crystallization front is described by diffusion equation:

$$\frac{\partial C_{\mathrm{Mg}}}{\partial t} = D\frac{\partial^2 C_{\mathrm{Mg}}}{\partial x^2} \quad (13)$$

with initial and boundary conditions:

$$\begin{aligned} C_{\mathrm{Mg}} &= C_1^{\mathrm{L}}, \qquad x \geq 0, \qquad t = 0, \\ \frac{\partial C_{\mathrm{Mg}}}{\partial x} &= -J_0/D, \qquad x = 0, \qquad t \geq 0. \end{aligned} \quad (14)$$

The solution of this problem is the following:[10]

$$\Delta \equiv \frac{(C_{\mathrm{Mg}} - C_1^{\mathrm{L}})}{(C_1^{\mathrm{L}} - C_1^{\mathrm{S}})} = \frac{2V}{D}\{(\frac{Dt}{\pi})^{1/2} e^{-x^2/4Dt} - \frac{x}{2} erfc(\frac{x}{2\sqrt{Dt}})\}. \tag{15}$$

The concentration increases with time. The maximum concentration excess is at the crystallization front (x=0):

$$\Delta_{\max} \equiv \frac{(C_{\mathrm{Mg}}|_{x=0} - C_1^{\mathrm{L}})}{(C_1^{\mathrm{L}} - C_1^{\mathrm{S}})} = 2V(\frac{t}{\pi D})^{1/2} \tag{16}$$

The function (Supplementary Equation 15) is shown in Supplementary Figure 8. As can be seen, the relative Mg concentration excess, Δ/Δ_{max}, decreases with the distance from the crystallization front; for example, at the distance $x = \sqrt{Dt}/4$ it equals $\Delta \approx 0.8\Delta_{\max}$(that will be used below as a higher concentration zone), at $x = \sqrt{Dt}/2$ it equals $\Delta \approx 0.6\Delta_{\max}$ $\Delta \approx 0.6\Delta_{max}$, and at the distance $x = \sqrt{Dt}$ it equals $\Delta \approx 0.35\Delta_{\max}$.

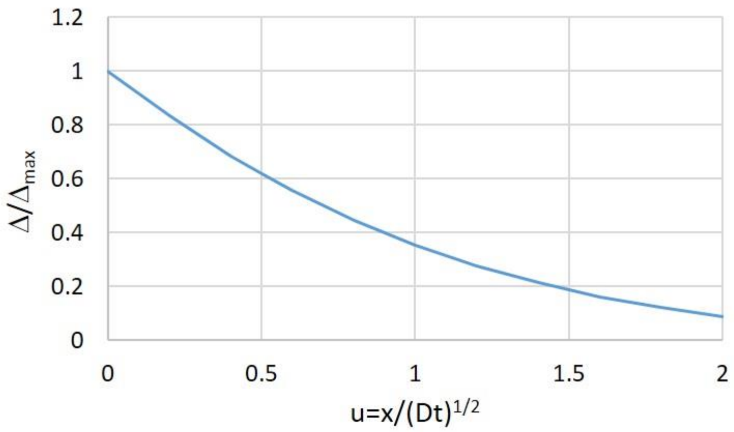

Supplementary Figure 8. *Relative increase of Mg concentration ahead of the crystallization front as a function of distance from the crystallization front.*

At a certain moment, the concentration in the diffusion zone $x \le \sqrt{Dt}/4$ may exceed the critical value, when the Mg-ACC matrix becomes unstable against spinodal decomposition, and a new secondary spinodal decomposition will start in this diffusion zone. It will result in the formation of new Mg-rich nanodomains and Mg-ACC matrix with concentration C_1^{L}. The advancement of the crystallization front forms a new calcite layer with higher density of Mg-rich nanodomains and as a result, with increased

concentration of Mg. In order to reach the critical Mg concentration ahead of the crystallization front, ~(15÷16)%, the maximum Mg excess should reach the values of several atomic percent, and, if we assume reasonable values of $(C_1^{\mathrm{L}} - C_1^{\mathrm{S}}) \sim (2 \div 4)\%$, then, $\Delta_{\max}$~1. Using Supplementary Equation 16, one can find the required time:

$$\bar{t} = \frac{\pi D}{4V^2} \tag{17}$$

and the corresponding widths of the layers of lower and higher Mg concentration:

$$\Delta L_{12} = V\bar{t} = \frac{\pi D}{4V} \ , \tag{18}$$

$$\Delta L_{23} = \frac{\sqrt{D\bar{t}}}{4} = \frac{\sqrt{\pi}}{8}\frac{D}{V} = \frac{\Delta L_{12}}{2\sqrt{\pi}} \tag{19}$$

Using reasonable values for diffusion coefficient of Mg in gel/liquid ACC, $D \approx 10^{-15}\mathrm{m^2.s^{-1}}$ and crystallization rate $V \approx 0.001$ µm.s^{-1}, yields $\Delta L_{12} \approx 0.78$ µm, $\Delta L_{23} \approx 0.22$ µm, which are close to the observed parameters of layered structure in the brittle stars.

We would like to note that the two presented models for the formation of the layered structure involve crystallization rates of different order of magnitude (0.01 and 0.001 µm.s^{-1}, respectively). The latter is unknown, the first model would corresponds higher crystallization rate, and the second would correspond to lower crystallization rate.

Supplementary References

1. Busenberg, E. & Niel Plummer, L. Thermodynamics of magnesian calcite solid-solutions at 25°C and 1 atm total pressure. *Geochim. Cosmochim. Acta* **53**, 1189–1208 (1989).

2. Zolotoyabko, E. *et al.* Differences between Bond Lengths in Biogenic and Geological Calcite. *Cryst. Growth Des.* **10**, 1207–1214 (2010).

3. Bhattacharyya, S. & Abinandanan, T. A. *A study of phase separation in ternary alloys*. *Bull. Mater. Sci* **26**, (2003).

4. Uhlmann, D. R., Chalmers, B. & Jackson, K. A. Interaction Between Particles and a Solid-Liquid Interface. *J. Appl. Phys.* **35**, 2986–2993 (1964).

5. Rempel, A. W., Wettlaufer, J. S. & Worster, M. G. Premelting dynamics in a continuum model of frost heave. *J. Fluid Mech.* **498**, 227–244 (2004).

6. Aubourg, P. F. Interaction of second-phase particles with a crystal growing from the melt. (1978).

7. Saint-Michel, B., Georgelin, M., Deville, S. & Pocheau, A. Interaction of Multiple Particles with a Solidification Front: From Compacted Particle Layer to Particle Trapping. *Langmuir* **33**, 5617–5627 (2017).

8. Vand, V. Theory of viscosity of concentrated suspensions. *Nature* **155**, 364–365 (1945).

9. Polishchuk, I. *et al.* Coherently aligned nanoparticles within a biogenic single crystal: A biological prestressing strategy. *Science (80-.).* **358**, 1294–1298 (2017).

10. Carslaw, H. S. & Jaeger, J. C. Conduction of heat in solids. *Oxford Clarendon Press. 1959, 2nd ed.* (1959).
